# RhizoBindingSites, a Database of DNA-Binding Motifs in Nitrogen-Fixing Bacteria Inferred Using a Footprint Discovery Approach

**DOI:** 10.3389/fmicb.2020.567471

**Published:** 2020-11-05

**Authors:** Hermenegildo Taboada-Castro, Jaime Abraham Castro-Mondragón, Alejandro Aguilar-Vera, Alfredo José Hernández-Álvarez, Jacques van Helden, Sergio Encarnación-Guevara

**Affiliations:** ^1^Center for Genomic Sciences, National Autonomous University of Mexico, Cuernavaca, Mexico; ^2^Centre for Molecular Medicine Norway (NCMM), Nordic EMBL Partnership, University of Oslo, Oslo, Norway; ^3^CNRS, IFB-core, UMS 3601, Institut Français de Bioinformatique, Évry, France; ^4^Laboratoire Theory and Approaches of Genome Complexity (TAGC), Inserm, Aix-Marseille Univ, Marseille, France

**Keywords:** DNA binding motif, nitrogen-fixing bacteria, footprinting discovery, database, transcriptional regulation

## Abstract

Basic knowledge of transcriptional regulation is needed to understand the mechanisms governing biological processes, i.e., nitrogen fixation by Rhizobiales bacteria in symbiosis with leguminous plants. The RhizoBindingSites database is a computer-assisted framework providing motif-gene-associated conserved sequences potentially implicated in transcriptional regulation in nine symbiotic species. A dyad analysis algorithm was used to deduce motifs in the upstream regulatory region of orthologous genes, and only motifs also located in the gene seed promoter with a *p*-value of 1e-4 were accepted. A genomic scan analysis of the upstoream sequences with these motifs was performed. These predicted binding sites were categorized according to low, medium and high homology between the matrix and the upstream regulatory sequence. On average, 62.7% of the genes had a motif, accounting for 80.44% of the genes per genome, with 19613 matrices (a matrix is a representation of a motif). The RhizoBindingSites database provides motif and gene information, motif conservation in the order Rhizobiales, matrices, motif logos, regulatory networks constructed from theoretical or experimental data, a criterion for selecting motifs and a guide for users. The RhizoBindingSites database is freely available online at rhizobindingsites.ccg.unam.mx.

## Introduction

Biological nitrogen fixation is a byproduct of beneficial symbiosis between some species of alpha- (order Rhizobiales) and beta-proteobacteria and leguminous plants. The promotion of biological nitrogen fixation in crops will protect against the major impacts of global warming: it substitutes the chemical fertilization of crops, reducing pollution and recycling excess atmospheric CO_2_. In addition, it provides basic grains for human consumption and pasture for animal breeding.

There is limited experimental information on transcriptional regulation in the Rhizobiales order; one strategy is to extend experimental regulatory motifs in bacterial genomes with bioinformatic tools (see below). However, the information is insufficient for answering basic questions, such as how the genomic circuitry of transcription factors (TFs) is in proteomic profiles.

The high number of genomes sequenced from *Rhizobium* species eases the inference of transcriptional regulatory networks (TRNs). Given that regulatory interactions between transcription factors and their target genes are well conserved in closely related organisms (Mushegian and Koonin, 1996) and that transcriptional regulation in bacteria occurs mainly in the upstream regions of genes, it is possible to infer common regulators (i.e., TF binding motifs) by combining the upstream sequences of orthologs of a given gene in a given taxon (e.g., the upstream sequences of the orthologs genes of LexA in the Enterobacteriales); this approach is known as phylogenetic footprinting (McCue et al., 2001; Defrance et al., 2008; Janky and van Helden, 2008). The discovered motifs can then be used for the *in silico* detection of transcription binding sites (TFBSs) when a binding site (BS) is from a TF or simply a BS in regard to a non-TF gene. We use TFBS to refer to all motifs located in the upstream regions of genes or operons, and this information can be used to build TRNs or to infer regulons (groups of genes regulated by the same TF).

Phylogenetic footprinting has been successfully used to complement TRNs in other bacteria, such as *Escherichia coli* K12 (McCue et al., 2001; Freyre-González et al., 2008), and is particularly useful when the studied TF binding motif is well conserved across a group of related organisms. It must be noted that one key point in the applicability of phylogenetic footprinting in bacterial genomes is the organization of bacterial genomes into operons and the general lack of distal regulation (e.g., enhancers).

Variants of a phylogenetic footprinting method have been used to construct databases with experimental transcriptional regulation and extended data (motifs deduced *in silico* from experimental data) of *Rhizobium* species, such as RhizoRegNet^1^, which is based on published regulatory interaction data (Krol et al., 2011). PRODORIC v.8.9^2^ contains a collection of TFBSs with experimental evidence from the literature (Grote et al., 2009). RegPrecise^3^ is a program based on an expanded reference collection of manually curated regulons (Novichkov et al., 2013). RegTransBase^4^ captures the knowledge from published scientific literature for the verification of predictions (Cipriano et al., 2013). However, for *Rhizobium* species, only a few TFs with motifs are known, and these databases contain limited genomic information and cannot infer regulons from co-expressed proteins, which are groups of co-regulated operons of the response system in bacterial cells (McGuire et al., 2000; Liu et al., 2016).

To complement the missing information, we developed the RhizoBindingSites database^5^, a computational genomic framework of transcriptional regulation for nine symbiotic species of the order Rhizobiales that provides information for inferring transcriptional regulation in bacteria for better experimental designs on transcriptional regulation.

The RhizoBindingSites database includes information about numerous organisms, namely, *Rhizobium etli* CFN42, *R. etli* Mim1, *Bradyrhizobium diazoefficiens* USDA110, *Sinorhizobium fredii* NGR234, *Sinorhizobium meliloti* 1021, *Rhizobium leguminosarum* bv. *viciae* 3841, *Bradyrhizobium* sp. BTAi, *Azorhizobium caulinodans* ORS 571 and *Mesorhizobium japonicum* MAFF303099, and covers a great number of host plants in symbiotic relationships.

## Materials and Methods

The motif analysis was performed using Regulatory Sequences Analysis Tools (RSAT) in a Linux environment (Medina-Rivera et al., 2015; Nguyen et al., 2018)^6^.

### Motif Discovery

Position-specific scoring matrices (PSSMs), hereafter called motifs, were identified using the program RSAT for footprint discovery (Janky and van Helden, 2008). Briefly, the input for this program includes the name of an organism, one or more gene names and the name of a taxon (see the command in Appendix A). The program searches the orthologous genes in the given taxon for each gene of the desired organism with a best bidirectional hit and an *E*-value threshold 1.0e-5. For each selected ortholog, the program obtains the upstream sequences (−400 to −1) relative to the translation start site. These sequences are masked in the redundant fragments, and a motif discovery algorithm is applied to these sequences using dyad-analysis in RSAT (van Helden et al., 2000). This program looks for overrepresented variable-spaced motifs, which is the case for most bacterial TFs that bind DNA as dimers. The detected dyads are assembled into PSSMs. For this analysis, we used all the upstream sequences of all the organisms belonging to the order Rhizobiales as a background model (Brohée et al., 2011).

### Selection of Motifs and Genomic Scan

Given that the motifs were discovered from the promoters of the orthologs of each gene, it is possible that some discovered motifs do not exist in the organism of interest. Some of the discovered motifs are likely to be artifacts (i.e., motifs reflecting the taxon-wise nucleotide frequencies and that do not correspond to a *bona fide* TF binding motif from the gene seed). Then, for selection of relevant motifs, matrices representing the motifs were used to scan the promoter regions of their gene seeds using the RSAT program matrix-scan (Defrance et al., 2008; Thomas-Chollier et al., 2008; command in Appendix B). We selected the motifs predicting a TFBS in the gene seed promoter with a *p*-value ≤ 1e-4, where the *p*-value evaluates the weight of the site. The weight is obtained by dividing the probability of finding a sequence segment given the matrix and the probability of generating the sequence segment given the background model, according to the probabilities described in Staden (1989) and Bailey and Gribskov (1998).

For each gene, the selected motifs were used to scan all the regulatory regions of all the genes of a corresponding genome, this process was repeated for the nine genomes. The regulatory regions were located from −400 to −1 relative to the ATG (translation initiation site) of each gene, removing overlapping coding regions when required. We kept the predicted TFBSs with a *p*-value ≤ 1e-4, and we used all the upstream sequences of all the organisms belonging to the order Rhizobiales to do the background model.

### Matrix-Scan Data

Predicted binding sites were divided according to the level of confidence, i.e., low (1.0e-4 to 9.9e-4), medium (1.0e-5 to 9.9e-5), and high (1.0e-6 to lowest data) *p*-values, to emphasize the importance of data with a low *p*-value. This scheme was based on the rationale that the homology between the sequence of the matrix and the segment of DNA of the regulatory region with the same matrix size is higher for low *p*-values than for high *p*-values. Predicted binding sites for each gene are reported in the motif information window, showing the motif in both strings for each gene.

### Motif Information

**FIGURE 1:**
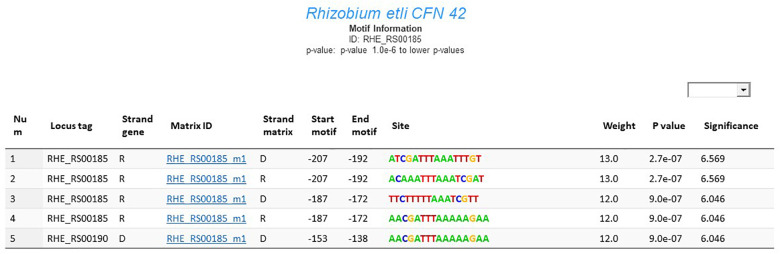
Motif information window for the gene RHE_RS00185 from *R. etli* CFN42.

The motif information window in RhizoBindingSites contains data from a genomic matrix-scan analysis with the matrices from each gene. These data represent an expected regulon that will hereafter be referred to as an “e-regulon” to distinguish it from the regulon deduced experimentally. These genes sharing one or more motifs from a gene in their upstream promoter region are shown; the presented information includes the locus tag of the gene; strand of the gene location; associated motif, which is identified by the locus name or locus tag of the gene followed by the letter “m” with the number of the matrix (i.e., RHE_RS30790_m1); strand of the motif in the upstream region; start/end position of the motif; sequence of the site motif; and weight, *p*-value and significance for the site (Figure 1; Medina-Rivera et al., 2015; Nguyen et al., 2018).

### Gene Information

**FIGURE 2:**
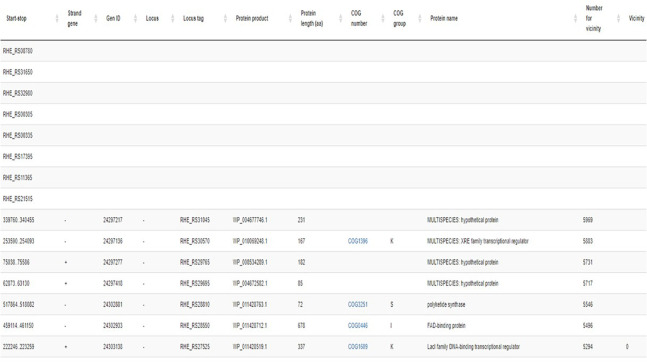
Gene information window for the gene RHE_RS00185 from *R. etli* CFN42.

The gene information window in the RhizoBindingSites database shows unique genes from an e-regulon (Figure 2). It includes the identifier locus tag (when there is no function annotated) or the start-stop position sequence of the gene, gene strand location, gene ID, name of the locus, identifier locus tag, protein product identifier, number of amino acids in the protein, COG number, COG group, and number of the gene in the genome; an equal number is used for genes near the three genes according to the number of the genes in the genome (see below), and the total number of unique genes is included at the bottom of column 1 (Figure 2). Note that some genes lack functional information although they are grouped according to shared motifs.

### Vicinity of Genes Sharing Motifs

In bacterial genomes, genes that are proximal to each other are often functionally related, for example, organized in the same operon and regulated by the same TF(s) (Strong et al., 2003; Bowers et al., 2004; Rodriguez-Llorente et al., 2009; Pannier et al., 2017). Neighborhood analysis was performed to detect genes within three genes of an e-regulon. First, all genes of a whole genome were enumerated starting from the replication origin. Second, unique genes from an e-regulon were sorted in decreasing order. Finally, new progressive numeration starting from zero was applied (Figure 2, column 12) to groups of pairs of genes within three genes. For genes organized in an operon, this criterion of vicinity should be extended per operon.

### Motif Conservation in the Taxon Rhizobiales

All methods for predicting TFBSs produce false positives. To determine how conserved motifs are in the taxa and gain more confidence in these data, the RhizoBindingSites database makes it easier for users to search for predicted motifs in the upstream sequences of orthologous genes in the order Rhizobiales. Detection of conserved motifs across taxa was performed using the RSAT command matrix-scan (command Appendix C; Figure 1; Medina-Rivera et al., 2015; Nguyen et al., 2018).

### Matrix Quality Analysis

We selected fifteen TFs with the highest coverage from each genome, and their respective matrices were analyzed with the program matrix-quality (Medina-Rivera et al., 2011). Matrices were run on the sequences from which the matrix was deduced, and a control was constructed by the program with the leave-one-out (LOO) procedure, which consists of discarding one site of the collection of sites that were used to construct the matrix. Then, the matrix was rebuilt with these sites, and sensitivity was newly assessed against the collection of sequences used originally. Most of our data were similar to the LexA transcriptional regulator (Medina-Rivera et al., 2011), showing a clear difference between theoretical and empirical score distributions with LOO control and the original matrices (data not shown).

### Comparison of Reported and Predicted Data of Transcriptional Regulation

We searched for coincidence of the published TF target genes in experimental transcriptional regulation data from *R. etli* CFN42, *S. meliloti* 1021, *B. diazoefficiens* USDA110, *R. leguminosarum* bv. *viciae* 3841, and *S. fredii* NGR234 taken from the RegTransBase database (Cipriano et al., 2013). We used the target genes of the predicted TFBSs to build putative regulons, which were then compared against the predicted data from RhizoBindingSites database (Supplementary Table 9).

### Genomic Coverage

Genomic coverage is the percentage of genes of an e-regulon with respect to the total number of genes from e-regulons (Figure 2, column 1) for each *p*-value category (Supplementary Tables 2–4, column 5). Then, for each gene of *R. etli* CFN42, respective orthologous genes were paired (column 1), and information on the function of each gene was added (columns 6 and 7).

### Comparing Genomic Coverage Between Orthologs

Genomic coverage of each gene of *R. etli* CFN42 and its respective orthologs for each species were used (Supplementary Tables 2–4, column 5) to produce a chart, and the correlation coefficient was calculated for each pair of strains for each *p*-value (Supplementary Tables 5–8).

### TFs With Vicinity

Genes with vicinity involved in transcriptional regulation grouped in Cluster orthologous Group K (COGK) (Tatusov et al., 2003) (TFs, response regulators, two-component response regulators, sigma factors, and anti-sigma factors) were analyzed for each genome in the three categories of *p*-values from the gene information window (Figure 2, column 9). We obtained 79% COGK annotated genes per genome on average (Supplementary Table 10).

### Some Examples of How to Use the RhizoBindingSites Database for a Computational Study

#### An Inferred Transcriptional Regulatory Network for the Synthesis of a Nodulation Factor From *R. etli* CFN42

We carried out an *in silico* search to determine how the genes involved in the synthesis of a nodulation factor are regulated. It is recognized that the transcriptional regulator *nodD* activates the transcription of genes for the synthesis of the nodulation factor (Rossen et al., 1985; Laranjo et al., 2014). The nodulation factor excreted to the rhizosphere promotes the first changes in *Phaseolus vulgaris* roots to establish symbiosis with *R. etli* CFN42 (Winsor, 1989; Cárdenas et al., 1995; Wang et al., 2018). A list of genes annotated for the synthesis of the nodulation factor was made according to the annotation of the genome of *R. etli* CFN42^7^, considering that some of them may be inside of an operon and that the regulatory region of this operon is from the first gene (Figure 5 and Supplementary Table 11).

#### How to Find an Expected Transcriptional Regulator of the Non-TF Gene RHE_RS00040 in the e-Regulon of RHE_RS00040

In the output from matrix-scan, the TF for the transcriptional regulator of the corresponding non-TF gene can be included. The non-TF gene RHE_RS00040 is used as an example, and the process is described in the user guide. The instructions are as follows: (1) Navigate to the *R. etli* CFN42 genome; (2) Navigate to the Gene Information section; (3) Introduce the gene example, and select a *p*-value of 1e-6 or lower; (4) Download the corresponding file in tsv format, open it with gedit, make a copy, and paste the copy into an Excel spreadsheet; (5) Select and sort the columns by COG group, and copy all the transcriptional regulators (nine transcriptional regulators); and (6) Paste the transcriptional regulators into the left box of the “Prediction of regulons” application, type the RHE_RS00040 locus into the right box, and download the data.

#### How the TFs of the Matrix-Scan Output of the Non-TF Gene RHE_RS00040 Can Be Interregulated

We observed some TFs in the output matrix-scan data. As an example of how to use these data, the output matrix-scan data of the non-TF gene RHE_RS00040 were taken to assess how the TFs could be interregulated. The process is described in the user guide as follows: (1) Navigate to the *R. etli* CFN42 genome; (2) Navigate to the Gene Information section; (3) Introduce the gene sample, and select a *p*-value of 1e-6 or lower; (4) Download the file in tsv format, open it with gedit, make a copy, and paste the copy into an Excel spreadsheet; (5) Select and sort the columns by COG group, and copy all the transcriptional regulators (nine transcriptional regulators); and (6) Paste the transcriptional regulators into both the left and right boxes of the “Prediction of regulons” application, and press “view network graph.”

## Results and Discussion

RhizoBindingSites (see text footnote 5) is a database of predicted DNA-binding motif regulatory regions associated with TF and non-TF genes from nine genomes of Rhizobiales (consult the user guide in the RhizoBindingSites database). Presumption of a motif in the gene seed may help avoid detecting motifs by chance and raise the possibility of finding genes involved in the gene regulatory process. The goal of RhizoBindingSites is to provide TF and non-TF binding motifs and gene information for each gene at three levels of confidence and to facilitate motif conservation analysis in the order Rhizobiales. In addition, the user can obtain the motif logo and regulons from predicted or experimental data.

Regulatory sequences analysis tools footprint discovery data showed that in the nine genomes, 62.7% of genes had 4.6 matrices per gene on average. Genes with motifs accounted for an average of 80.44% of genes, with 19613 matrices per genome (Supplementary Table 1). Genes without motifs may be due to a limited size of the upstream region, the absence of orthologs in the order Rhizobiales, the lack of a promoter (i.e., genes organized in operons) or non-significant dyads (Nguyen et al., 2018).

The ten genes with the highest genomic coverage were taken from the matrix-scan output of the *R. etli* CFN42 genome at *p*-value levels of 1e-4, 1e-5, and 1e-6. On average, 3324, 940, and 219 genes were covered at the three levels of *p*-values, respectively (data not shown). This means that the alignment of the matrix sequence and the upstream region (−400 to −1) of genes at a *p*-value of 1.0e-4 to 9.9e-4 is acceptable with a low identity, probably including more false positives than at the medium level of restriction (*p*-value of 1.0e-5 to 9.9e-5) or even the stricter level (*p*-value of 1.0e-6 or lower) in the *R. etli* CFN42 genome. This is a general observation for all genomes in this study. Accordingly, the RSAT matrix-scan program output data had the lowest *p*-value per matrix (Thomas-Chollier et al., 2008). Motifs with a *p*-value of 1.0e-4 may be relevant for relaxed consensus promoters, such as *sigA* gene from *R. etli* CFN42 (Ramírez-Romero et al., 2006).

It is worth noting that the motif information window contains predicted motifs of TF and non-TF genes. For matrix-scan data of non-TF genes, the genomic matrix-scan output contains all genes sharing one or some motifs of a gene, based on the principle that most of the genes that encode enzymatic or structural proteins have orthologs and conserved sequences between them, probably involved in the transcriptional regulation of the gene. It is very likely that the gene and its transcriptional regulator share similar sequences, such that the transcriptional regulator may be part of the e-regulon of the gene. A new search with matrices of this TF is recommended to infer the regulation of these genes (Figure 1). There is an example of how to search for a hypothetical TF from the e-regulon of the non-TF gene RHE_RS00040 in the user guide, while a genomic matrix-scan output from motifs of a TF suggests a functional association. Moreover, for genes without a deduced matrix, in the motif information window, there is an option for searching for TF matrices that recognize a binding site in the upstream regions of those genes, particularly when they do not have sufficient orthologs in the order Rhizobiales but have a sufficient sequence in its promoter region (Figure 1).

To determine the conservation of the genomic coverage number between orthologous genes from all genomes (Supplementary Tables 2–4 column 5), we used genomic coverage data per gene to calculate correlation coefficients at the three *p*-value levels between *R. etli* CFN42 and the orthologs of the eight strains. In addition, we compared *S. meliloti* 1021 with *S. fredii* NGR234 1021, which is more closely related than *R. etli* CFN42 to *S. meliloti* and that *R. etli* CFN42 to *S. fredii* NGR234 (Black et al., 2012; Supplementary Tables 5–8). The correlation coefficient is high when strains are closely related in a phylogenetic tree with a 16S ARN (Black et al., 2012). These data showed that the genomic coverage was similar between phylogenetically related species but different among distant phyla.

The users may choose the *p*-value for motif prediction in the motif information window from the RhizoBindingSites database (Figure 1). After exploring possible functional data, it is recommended to select data with the lowest *p*-value level, since the *p*-value evaluates the weight of the site (see “Materials and Methods”). Moreover, for better selection of predicted data, it is necessary to consider the following in the gene information window of RhizoBindingSites (Figure 2): the function of the query protein, the COG classification (Galperin et al., 2015), the preponderancy of functions in the group of genes sharing the same motif (see the chart located in the gene information window), and other TFs considering vicinity (Bowers et al., 2004; Figure 2, column 12). It is worth noting that the user will have a set of genes per genome sharing a motif from a TF from the RhizoBindingSites database or an experimental profile of proteins with co-expressed TFs. In those cases, the presence of the regulons can be inferred with the application “Prediction of regulons” (located in the motif information window for each species). Additionally, the user may enrich their data by searching for the genomic context of the query and target genes on the Gene Context Tool NG v3 website at http://bioinfo.ibt.unam.mx/gecont (Martinez-Guerrero et al., 2008).

**FIGURE 3:**
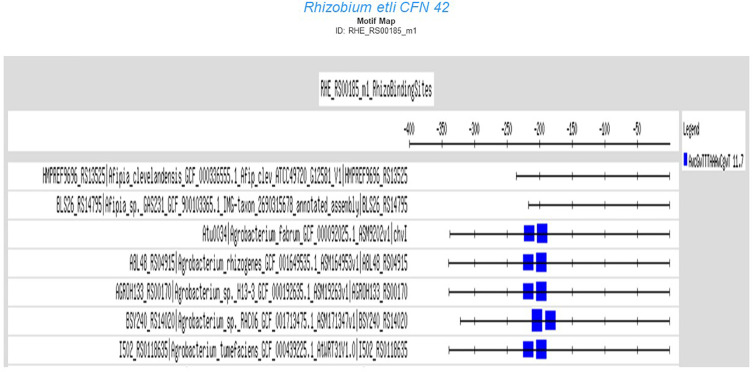
Motif map of the RHE_RS00185_m1 matrix from *R. etli* CFN42 in orthologs from taxon Rhizobiales.

**FIGURE 4:**
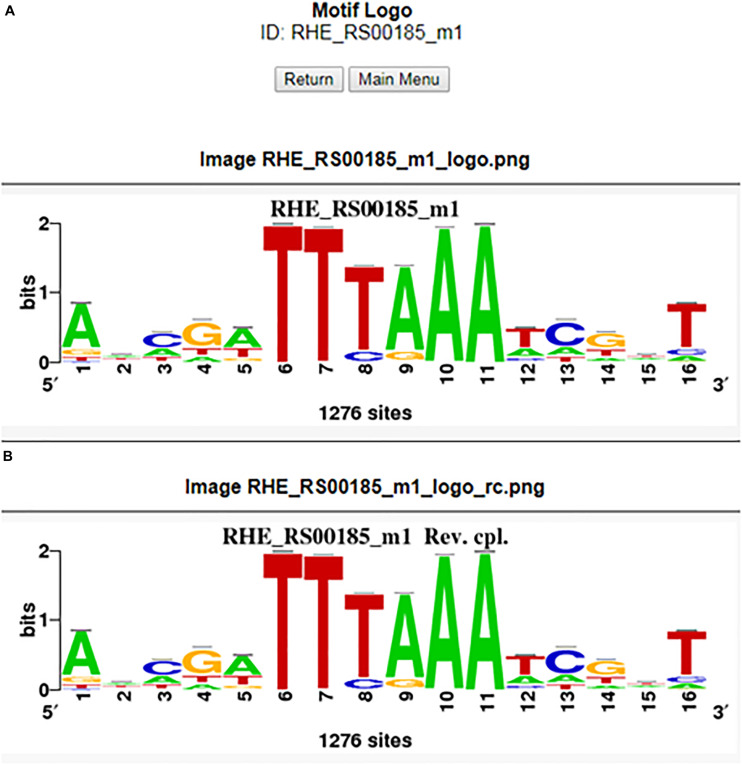
Motif logo from the RHE_RS00185_m1 matrix for the forward **(A)** and reverse **(B)** complementary strands.

The user may be able to determine how conserved a motif is in the order Rhizobiales by conducting a matrix-scan analysis (Thomas-Chollier et al., 2008), specifically, by clicking the motif map button in the motif information window of the RhizoBindingSites database. This option shows a matrix-scan analysis with the matrix selected and upstream sequences (−400 to −1) of orthologs of the gene query. The output is a graph (.png file) showing a list of orthologs from the gene query with boxes into the upstream −400 to −1 putative promoter region depicted by a line. This line contains colored boxes representing the motifs, with size indicating the motif score. The box is above the line if the binding site is on the direct strand (D) or below if it is on the reverse strand (R) (Figure 3). Note that when the motif is conserved, the binding site is mapped in the same orientation and same position in phylogenetically related groups and may or may not be in a different position between groups within the order Rhizobiales, meaning that the motifs are not predicted by chance (Figure 3). In addition, the RhizoBindingSites database has a window where the user can obtain a logo from the motif on the D and R strands by clicking on the motif logo button (Figures 4A,B), as with RSAT convert-matrix 2004 (Crooks et al., 2004; Medina-Rivera et al., 2015).

**FIGURE 5:**
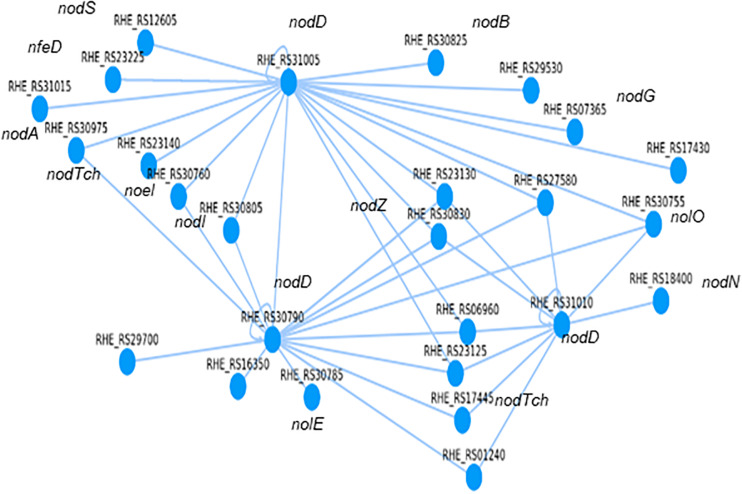
Computational regulatory network for the synthesis of the nodulation factor from *R. etli* CFN42.

We compared predicted data with experimental data collected from the literature in the RegTransBase database (Cipriano et al., 2013). We found five hits of seven regulons from *R. etli* CFN42 matrix-scan reported data: twenty-six of twenty-seven from *S. meliloti* 1021, nine of twelve from *B. diazoefficiens* USDA110, nine of ten from *R. leguminosarum* bv. *viciae* 3841 and the unique regulon described from *S. fredii* NGR234 (Supplementary Table 9). For the sequence and position of the predicted motif, our data should be highly approximate relative to experimental data because the motifs are predicted by positional consensus of nucleotides from orthologs for each gene. This was shown for the predicted transcriptional regulator NodD motif from *R. etli* CFN42 (Meneses et al., 2017). Thus, the predicted data showed great correspondence with previously reported data, despite the limited number of TFs with experimental information on transcriptional regulation per genome from nitrogen-fixing symbiotic species (Cipriano et al., 2013). Moreover, the capacity of a matrix to find motifs with the matrix-quality program from TFs with the highest genomic coverage was determined, and it showed great sensitivity (Medina-Rivera et al., 2011) (data not shown).

Additionally, in the matrix-scan output data, we searched for nearby COGK genes for the three levels of *p*-values (“Materials and Methods”). The data showed that for six genomes (*R. etli* CFN42, *R. etli* Mim1, *S. fredii* NGR234, *B. diazoefficiens* USDA110, *B.* sp. BTAi1, and *M. japonicum* MAFF303099), the percentage of nearby COGK genes was higher at *p*-values 1.0e-6 or lower than at *p*-values of 1.0e-4 to 9.9e-4 and 1.0e-5 to 9.9e-5 (Supplementary Table 10). This suggests higher specificity for data at a more restrictive *p*-value of 1.0e-6 or lower. Additionally, we confirmed that our data were not predicted by chance, since the average of ten genes with the greatest genomic coverage was 15.1 times higher at *p*-values of 1.0e-4 to 9.9e-4 than at *p*-values 1.0e-6 or lower (see above). These neighboring genes may be co-regulated, and since they share a motif, they may be tightly co-regulated and highly expressed (Pannier et al., 2017).

As examples of how to use the RhizoBindingSites database, we have included three *in silico* studies. The first example was to determine the transcriptional regulators involved in the synthesis of a nodulation factor from *R. etli* CFN42. This analysis was performed with the option “auto,” which selects the TF gene-target relationship with the lowest *p*-value (see “Materials and Methods” and Guide for Users). These data showed that the three *nodD* transcriptional regulators of type lysR were involved in the synthesis of the nodulation factor, revealing that eleven genes were regulated for more than one *nodD* transcriptional regulator. RHE_RS31005 *nodD* was located at the top of the network, and there was interregulation between the two *nodD* transcriptional regulators RHE_RS31005 and RHE_RS30790 and between RHE_RS30790 and RHE_RS31010 (Figure 5 and Supplementary Table 11). A second study consisted of using the matrix-scan data from a non-TF gene to search for the potential TFs involved in the regulation of the non-TF-gene RHE_RS00040, based on the principle that the non-TF gene and its transcriptional regulator should share a motif; consequently, they should be included in the matrix-scan output data (e-regulon). These data showed that among nine TFs, only the matrix RHE_RS26495_m3 of the TF RHE_RS26495 was involved in the transcriptional regulation of the non-TF gene RHE_RS00040. The process is detailed in the user guide (“Materials and Methods”). In the third example, since for matrix-scan output data from RHE_RS00040, a non-TF gene, nine TFs shared one or more motifs, transcriptional regulation was inferred only between these TFs, with the lowest *p*-value for a TF-TF relationship. As was hypothesized, a Cytoscape graph (Shannon et al., 2003) showed interregulation between the TFs, with the TF RHE_RS27920 located at the top of the network with the greatest number of connections. The analysis and graph are described in detail in the user guide (“Materials and Methods”).

The RhizoBindingSites database (see text footnote 5) provides bioinformatic information that may contribute to the design of experiments on the transcriptional regulation of nitrogen-fixing symbiotic species which covers a great number of host plants and is thus useful for inferring regulons and providing information for regulatory networks.

## Data Availability Statement

The datasets presented in this study can be found in online repositories. The names of the repository/repositories and accession number(s) can be found in the article/ Supplementary Material.

## Author Contributions

HT-C, SE-G, and JH conceived the idea. JH, JC-M, and HT-C designed the analysis. HT-C, SE-G, and JC-M analyzed the results and drafted the manuscript. AH-Á built the web server. AH-Á and AA-V contributed with the code. SE-G and JC-M revised the manuscript. All authors read and approved the final manuscript.

## Conflict of Interest

The authors declare that the research was conducted in the absence of any commercial or financial relationships that could be construed as a potential conflict of interest.

## Footnotes

1 http://rhizoregnet.cebitec.uni-bielefeld.de

2 http://www.prodoric.de

3 http://regprecise.lbl.gov

4 http://regtransbase.lbl.gov

5 http://rhizobindingsites.ccg.unam.mx

6 http://embnet.ccg.unam.mx/rsat/

7 https://www.ncbi.nlm.nih.gov/genome/?term=Rhizobium+etl

## Appendix A

Command for footprint discovery analysis of *Rhizobium etli* CFN42.

RSAT footprint-discovery was conducted with the following parameters: -org Rhizobium_etli_GCF_000092045.1_ASM9204v1 -taxon Rhizobiales -all_genes -sep_genes -lth occ 1 -lth occ_sig 0 -uth rank 50 -return occ,proba,rank -filter -bg_model taxfreq -task query_seq,filter_dyads,orthologs,ortho_seq,purge,dyads,maps,gene_index,index. This program deduced one to five motifs for each gene.

## Appendix B

Command for the genomic matrix-scan analysis of *Rhizobium etli* CFN42.

The following parameters were used: -matrix_format transfac -pseudo 1 -decimals 1 -2str -origin genomic –bginput background_model -markov 2 -bg_pseudo 0.01 -return limits -return sites -return pval -lth score 1 -uth pval 1e-4 -i -seq_format fasta upstream_sequences -n score (Thomas-Chollier et al., 2008).

## Appendix C

Matrix-scan command for analysis of conservation of a motif across the orthologs in Rhizobiales.

The parameters were as follows: matrix-scan -quick -return limits,sites,pval -decimals 1 -origin end -consensus_name -matrix_format transfac –m (address of matrix) -pseudo 1 -decimals 1 -2str -bg_pseudo 0.01 -lth score 1 -uth pval 1e-4 -n score -seq_format fasta -bginput -markov 1 -i (address of upstream ortholog sequences in fasta format) | $RSAT/perl-scripts/feature-map -title “Site map; Rhizobiales; Rhizobium_etli_GCF_000092045.1_ASM9204v1;

RHE_RS17305_Rhizobium_etli_GCF_000092045.1_ASM9204v1_Rhizobiales_taxfreq” -format png –scalebar -legend –score thick -mapthick 16 -mspacing 2 -mlen 600 –o (output in png format).
